# Microbial Content of “Bowl Water” Used for Communal Handwashing in Preschools within Accra Metropolis, Ghana

**DOI:** 10.1155/2016/2617473

**Published:** 2016-07-31

**Authors:** Patience B. Tetteh-Quarcoo, Isaac Anim-Baidoo, Simon Kwaku Attah, Bawa Abdul-Latif Baako, Japheth A. Opintan, Andrew A. Minamor, Mubarak Abdul-Rahman, Patrick F. Ayeh-Kumi

**Affiliations:** ^1^Department of Medical Microbiology, School of Biomedical and Allied Health Sciences, University of Ghana, Accra, Ghana; ^2^Department of Medical Laboratory Science, School of Biomedical and Allied Health Sciences, University of Ghana, Accra, Ghana; ^3^Department of Science Laboratory Technology, Accra Polytechnic, Accra, Ghana; ^4^Department of Immunology, School of Biomedical and Allied Health Sciences, University of Ghana, Accra, Ghana

## Abstract

*Objective*. This study aimed at determining the microbial content of “bowl water” used for communal handwashing in preschools within the Accra Metropolis.* Method*. Six (6) preschools in the Accra Metropolis were involved in the study. Water samples and swabs from the hands of the preschool children were collected. The samples were analysed and tested for bacteria, fungi, parasites, and rotavirus.* Results*. Eight different bacteria, two different parasites, and a fungus were isolated while no rotavirus was detected. Unlike the rest of the microbes, bacterial isolates were found among samples from all the schools, with* Staphylococcus* species being the most prevalent (40.9%). Out of the three schools that had parasites in their water, two of them had* Cryptosporidium parvum*. The fungus isolated from two out of the six schools was* Aspergillus niger*. All bacteria isolated were found to be resistant to cotrimoxazole, ciprofloxacin, and ampicillin and susceptible to amikacin and levofloxacin.* Conclusion*. Although handwashing has the ability to get rid of microbes, communal handwashing practices using water in bowls could be considered a possible transmission route and may be of public concern.

## 1. Introduction 

Provision of a bowl of water for handwashing in preschools is an intervention by childcare facilities in Ghana, to maintain hygienic practices and prevent transmission of microbes. In the year 2005, the Ghana School Feeding Programme (GSFP) was implemented as one of the most important social interventions by the government, to help boost public basic school enrolments [1]. The programme offers one meal each day for all government preschoolers and primary school children. This provision is to reduce hunger and malnutrition, increase school enrolment and attendance, and stimulate local food production. Similarly, many privately owned preschools provide meals for their pupils. This has therefore necessitated the provision of water for handwashing before and after meals. “Bowl water” (water in a basin/bowl) has been adapted to serve this purpose whenever the flow of running tap water is interrupted.

Handwashing has been regarded as a significant preventive measure against diarrhoeal diseases [2]. It is an effective [2], feasible [2–5], and cost-effective [6] means of preventing gastroenteric infection worldwide. Recently, there has been growing awareness of its importance not only as a diarrhoeal disease preventive measure [2, 7], but also as part of a wider public health effort to reduce the disease burden of acute respiratory infections worldwide [2, 8–10]. The importance of handwashing has also been underlined in a recent review of measures to control the spread of pandemic influenza [11]. The two leading causes of disease burden globally, respiratory infections and diarrhoeal diseases, are responsible for half of all childhood deaths each year [12]. Therefore, good and adequate handwashing practice is a prerequisite to disease control and child's survival [2, 13].

With reference to an appropriate handwashing procedure [14], communal handwashing from a bowl of water is not the best practice. A handwashing facility, even with soap, on a communal basis, where the same water is used by more than one person, does not constitute an adequate handwashing facility [15, 16]. This practice, especially when the water in the bowl is not changed frequently, could increase the exchange of microbes among the children when the goal is to get rid of them. Therefore, this study aimed at determining the microbial content of “bowl water” used for handwashing in preschools within Accra Metropolis. This will help in the understanding of the possible role that “bowl water” could play in the transmission of infectious agents. It will also help appreciate whether, in the absence of flowing tap water, washing from a bowl of water could serve as an alternative way of getting rid of most microbes among children.

## 2. Methods 

### 2.1. Study Site and Sample Collection

Preschools within Accra, the capital of Ghana, that either are under the Ghana School Feeding Programme or are private schools that provide meals for children were selected for the current study. To maintain anonymous identity of the schools and confidentiality, the schools were coded as SA, SB, SC, SD, SE, and SF. Sterile plastic bottles were used to aseptically collect 1000 mL each of water samples used for handwashing and rinsing. Three water samples were taken from each school; one sample was taken from their main source, such as water storage tanks, commonly called “poly tank,” tap, and well, and labelled as “source.” Another sample was taken from the basins containing water for handwashing and labelled as “soapy water” and a third sample was taken from the basin containing water for rinsing and labelled as “rinse.” After a brief interview using a questionnaire, swabs were obtained from three randomly selected pupils from each school, before they washed their hands and after handwashing. The collected samples (both the water and the swabs) were transported on ice packs, to the laboratories at the Departments of Medical Microbiology and Medical Laboratory Sciences, all of the School of Biomedical and Allied Health Sciences, University of Ghana, for examination.

### 2.2. Parasitological Examination

One hundred millilitres (100 mL) of each water sample was centrifuged at 5000 rpm for 10 minutes. The supernatant was discarded and the remaining sediments were resuspended and examined by the direct wet mount and concentration techniques [17, 18]. Briefly, a drop of the sediment was placed on two clean grease-free microscope slides. To one, a drop of iodine was added and both were covered with coverslips. Each slide was then examined under a light microscope for the presence of life forms of protozoans and helminths. Formol-ether concentration method was employed on the rest of the sediments [17, 18]. After concentration, three slides were made from each resuspended sediment. Two out of the three slides were stained, one with iodine and the other with modified Z-N stain, and the third was examined directly as wet mount using standard procedures [19].

### 2.3. Isolation, Enumeration, and Identification of Bacteria and Fungi

The water samples were well mixed and using disposable sterile loops (2 mm inside diameter) each of the samples was inoculated onto MacConkey agar (MA) and blood agar (BA) (Oxoid Ltd., Basingstoke, UK). The swabs were also inoculated (directly) onto MA and BA. The plates were incubated at 37°C for 24 hours. The number of colonies seen was counted using a colony counter and recorded as colony forming unit per gram (cfu/g) for swabs and colony forming unit per millilitre (cfu/mL) for water samples. Bacteria colonies were further subcultured onto MA and BA, to obtain pure cultures and primary identification done using colonial morphology. Further identification was done based on a number of procedures including microscopy, Gram staining, oxidation-fermentation tests, and other biochemical tests such as urease test, catalase test, citrate utilization test, indole test, and triple sugar iron test [20–23].

Similarly, the water samples and swabs were inoculated on Sabouraud agar (Oxoid Ltd., Basingstoke, UK) for fungal growth and subsequent identification using standard protocol [19].

### 2.4. Susceptibility Testing of Bacteria

Susceptibility test for identified bacteria was determined using a modified form of the Kirby Bauer method. The antimicrobials tested with their disk concentrations in micrograms (*μ*g) were gentamicin (15), amikacin (30), tetracycline (30), cotrimoxazole (25), cefotaxime (30), meropenem (10), ampicillin (10), ceftriaxone (30), chloramphenicol (30), cefuroxime (30), levofloxacin (5), and ciprofloxacin (5) (Oxoid Ltd., Basingstoke, UK). These antimicrobials are among the common drugs found on the Ghanaian market. Briefly, on the procedure for antibiotic susceptibility testing, the test organism was emulsified in peptone water until the suspension was comparable with 0.5 McFarland's standard. A sterile cotton swab was dipped into the suspension and drained by pressing the swab against the inside of the bottle. The swab was then used to streak the entire surface of a Mueller-Hinton agar plate (Oxoid Ltd., Basingstoke, UK). The moisture was allowed to be absorbed for at least 15 minutes and sterile forceps were used to apply the antibiotic discs to the surface of the agar plates. The plate was kept at 4°C for 4–6 hours, so that the antibiotic can diffuse on the agar media after which it was incubated at 37°C for 18–24 hours. Zone diameters around the antibiotic discs were measured and later classified as susceptible or resistant based on the Clinical Laboratory Standards Institute (CLSI) criteria [24]. Control strain used for the susceptibility test was* Escherichia coli NCTC 10418*.

### 2.5. Virological Examination

The water samples were tested for rotavirus using the ProSpecT*™* Rotavirus ELISA kit (Oxoid Ltd., UK). The process was carried out using the protocol designed by the manufacturer (Oxoid Ltd., UK). To ensure quality and accurate results, positive and negative controls were run for each test.

## 3. Results

A total of 6 preschools were involved in the study. Three water samples and six swabs were obtained from each preschool making a total of 54 samples for the investigation. Five (83%) of the preschools were privately owned while only one was government owned (Table 1). The schools had different stages, ranging from preschool to junior high school (JHS) (Table 1).

**Figure 1:**
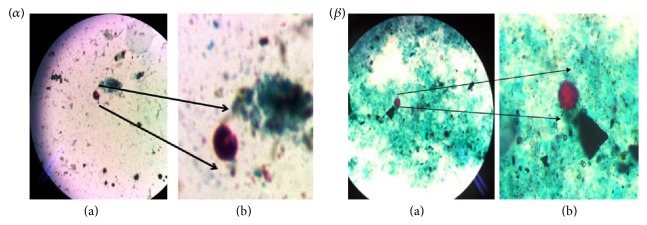
Modified ZN stained slides of parasites identified. (*α*) Oocysts of* Cyclospora cayetanensis*. (*β*) Oocyst of* Cryptosporidium parvum*. (a) represents magnification of ×1000 while (b) is a zoom-in of the oocyst.

Parasitological investigations identified two different parasites (Table 2).* Cryptosporidium parvum *and* Cyclospora cayetanensis* were both found in the rinsing water of school SB and the soapy water of school SD. Meanwhile,* Cryptosporidium parvum *was also found in the main water source of school SC (Figure 1).

Bacteriological investigations identified eight (8) different bacteria in the different water types (Table 2). The bacteria isolates were in the following proportions:* Staphylococcus *species (40.9%)*, Escherichia coli *(13.6%)*, Citrobacter *species (13.6%)*, Klebsiella *species (4.6%)*, Proteus mirabilis *(2.3%)*, Klebsiella pneumoniae *(9.1%)*, Salmonella *species (9.1%), and* Klebsiella oxytoca *(6.8%)*. Staphylococcus *specieswas the most common isolate from all the preschools except for SF.* Salmonella *species was isolated from four different preschools, while* Escherichia coli*,* Citrobacter* species, and* Klebsiella pneumoniae* were identified in three different schools.* Klebsiella oxytoca* and* Klebsiella *species were identified in two preschools and* Proteus mirabilis* was isolated from only one preschool.

Examination of the swabs showed that* Staphylococcus *species was present before handwashing and after handwashing.* Escherichia coli*,* Citrobacter *species,* Salmonella *species*, Klebsiella pneumoniae*, and* Klebsiella oxytoca* were only isolated from swabs obtained after handwashing (Table 2). Notably, just a few colonies were observed for most of the bacterial isolates, for the different sources of water and hand swabs. The ranges of colony forming units of the isolates from various samples are as follows:* Staphylococcus *spp. (1.2 × 10^2^–1.2 × 10^3^),* Salmonella *spp. (1.0 × 10^1^–3.2 × 10^1^),* E. coli *(1.1 × 10^1^–1.7 × 10^1^),* Citrobacter* spp. (1.4 × 10^1^–4.2 × 10^1^),* K. pneumonia* (1.5 × 10^2^–7.0 × 10^2^),* K. oxytoca *(2.0 × 10^2^–4.2 × 10^2^),* Klebsiella *spp. (1.0 × 10^2^–3.3 × 10^2^), and* P. mirabilis *(1.0 × 10^3^–1.5 × 10^4^). Apart from* Staphylococcus *spp. and* P. mirabilis, *all the remaining bacteria recorded cfu below 10^3^ (Table 3).

**Figure 2:**
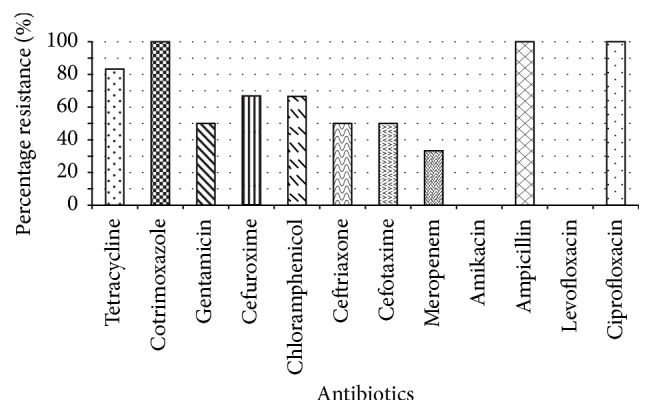
Percentage resistance of antibiotics tested. Six out of the eight isolates tested, namely,* Salmonella *species,* Citrobacter *species,* Escherichia coli, Proteus mirabilis, Klebsiella pneumoniae*,and* Klebsiella oxytoca. * The values of diameter (mm) measurement that were considered sensitive are as follows: tetracycline ≥ 26, cefotaxime ≥ 26, cotrimoxazole ≥ 24, ampicillin ≥ 17, gentamicin ≥ 20, ciprofloxacin ≥ 21, cefuroxime ≥ 18, meropenem ≥ 16, amikacin ≥ 20, chloramphenicol ≥ 30, ceftriaxone ≥ 21, and levofloxacin ≥ 17.

The isolated Gram negative bacteria were tested against twelve selected antimicrobial agents. All the organisms tested (6/6, 100%) were resistant to cotrimoxazole, ampicillin, and ciprofloxacin while they were susceptible (100%) to amikacin and levofloxacin. The organisms showed varying percentage resistance to the remaining antibiotics (Figure 2). Notably, for gentamicin, resistance was seen among* Salmonella *species,* Klebsiella pneumoniae*,and* Klebsiella oxytoca*. All bacteria, except for* Proteus mirabilis *and* Escherichia coli*,were resistant to cefuroxime. Similarly, resistance to chloramphenicol was observed in all the bacteria tested except for* Citrobacter *speciesand* Escherichia coli. * Meanwhile, the only organism susceptible to tetracycline was* Escherichia coli*.

**Figure 3:**
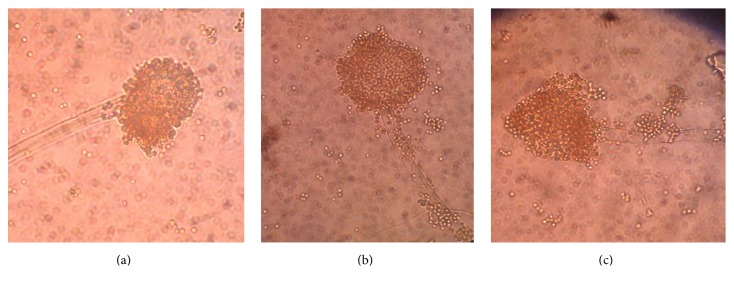
A wet preparation of* Aspergillus niger *showing “sporing structure.” ((a) and (b)) From hands of school children, (c) from soapy water.

From the fungal studies,* Aspergillus niger* was identified from hand swabs of school children from schools SB and SC before and after handwashing. The same fungal species was also found in the soapy water of school SB (Figure 3).

The rotavirus screening did not detect the presence of the virus in any of the water samples collected from the schools, as well as the swabs obtained from the hands of the children (Table 2). Other viruses could not be investigated due to inadequate resources.

## 4. Discussion

Bacteria isolates from the current study include* Salmonella *species*, Citrobacter *species*, Escherichia coli, Proteus mirabilis, Klebsiella pneumoniae*,and* Klebsiella oxytoca*. Most of these organisms have also been isolated from a work done by Tetteh-Quarcoo et al., when they investigated microbial carriage of cockroaches within the same geographical location as this study [25]. This shows likelihood of these bacteria species circulating within the metropolis. A recent study conducted by Ayeh-Kumi et al., on tiger nuts which were claimed to have been washed and sold in locations including that of the current study, also found bacterial isolates, including* Klebsiella oxytoca*, suggesting the association of this particular bacterium with water used within the metropolis [26]. Most of the bacteria isolated in this study are pathogenic or potentially pathogenic and are therefore of public health significance, especially when in connection with preschool children. For example,* Klebsiella pneumo niae* causes pneumonia, urinary tract infections, and wound infections [23, 24]. Studies have shown that individuals with recurrent infections and those with structural abnormalities of the urinary tract have an increased frequency of infection caused by bacteria such as* Klebsiella oxytoca* [27, 28].

The isolation of* Escherichia coli* shows that washing with bowl-water is a possible source of transmitting this bacterium, which causes acute diarrhoea, especially in children [29]. Although* Escherichia coli* is part of the normal flora of the intestinal tract, certain strains can cause moderate to severe gastroenteritis in adults and children [29].* Salmonella *species and* Escherichia coli* were isolated from even the source where the water used for handwashing was fetched. This is not surprising, since even sachet water, regarded as more purified, has been found to contain high levels of bacteria [30]. Almost all the bacteria isolated in this study are mostly responsible for enteric diseases in young children. All the same, the bacteriological methods used in this study provided not much quantification of bacterial load, unlike a study by Hoque et al. [31].* Staphylococcus *species was very common in each of the preschools. Infections from this organism occur when staphylococci enter the body through breaks, cuts, and abrasions in the skin or mucous membranes. Hence, children who have openings such as cuts and abrasions of the skin have a high risk of getting infection caused by this organism when they use such handwashing facilities. Isolation of* Citrobacter* species is a notable observation, since* Citrobacter *infections can be community acquired.* Citrobacter* species also cause meningitis, septicaemia, and pulmonary infections in neonates and young children. Most of these organisms were enteric bacteria which possibly found their way through accidentally, due to poor hygiene, suggestive of faecal contamination.

Observation from the antibiotic resistance testing of the current study conforms with the observation by Tetteh-Quarcoo et al. [25] and reemphasizes the assertion that some antibiotics such as cotrimoxazole, ampicillin, and ciprofloxacin have been on the Ghanaian market for a long time and therefore might have been subjected to indiscriminate use or abuse leading to the high levels of resistance recorded [32, 33]. Although ciprofloxacin and levofloxacin are both from the same class of antibiotics (fluoroquinolones), there was a notable difference in their performance against the bacteria isolates tested. This observation could be due to the probable indiscriminate use of ciprofloxacin in Ghana, since it is readily available compared to levofloxacin.* Citrobacter *species was found to be resistant to five out of the 12 antibiotics tested. In spite of this, Shih et al. [34] found that the combination of a beta-lactam and an aminoglycoside had better therapeutic results than a single agent alone for* Citrobacter* bacteremia; hence, children who acquire infection through handwashing can be treated using this recommendation.* Staphylococcus *species were isolated from swabs before handwashing and after handwashing.* Escherichia coli*,* Citrobacter *species,* Salmonella *species*, Klebsiella pneumoniae*, and* Klebsiella oxytoca* were only isolated from swabs obtained after handwashing. Another study also isolated* Staphylococcus *species,* Escherichia coli*, and* Klebsiella* from the hands of preschool nurses [35]. Most of the isolates found on the hand swabs were from the “after handwashing” samples and therefore it is suggested that the children might have picked the bacteria during handwashing process. All the same, it is noteworthy that just a few colonies were counted for most of the bacteria isolates. This implies that although pathogenic bacteria were among the agents isolated, infection from these organisms might mostly be manifested in immunocompromised children who use this handwashing process.

*Cryptosporidium parvum* and* Cyclospora cayetanensis* were present in some of the water samples used for handwashing in the preschools. These organisms are potential pathogens associated with water related diarrhoea outbreaks in healthy people and have devastating presentations in the immunocompromised individuals, particularly children [36]. The result of this study supports the findings of Obiri-Danso et al., when they carried out a study to determine the microbial quality of water on the streets of Kumasi [37]. These organisms are also notable for the stability of their oocysts in the environment [38]. Hence, the finding of these parasites in the “bowl water” used for handwashing by preschool children is of public health importance.

The current study identified* Aspergillus niger* from the hands of school children before and after handwashing and also from soapy water used for handwashing. This emphasizes the ubiquitous nature of this mould and its association with water [39, 40]. In a study, different species of fungi were recovered from Dead Sea water, many of which belonged to nonhalophilic terrestrial species, known for their diverse distribution [39]. Examples of these species include* Aspergillus niger *and* Cladosporium cladosporioides* [39]. In spite of their widespread occurrence, little attention has been given to the presence of fungi and their significance in preschool environments. The observation of* Aspergillus niger* from water samples used for handwashing suggests that the communal handwashing facility could be considered a possible transmission route forthis fungal species. Although* Aspergillus* species cause significant pulmonary infections,* Aspergillus niger* has rarely been reported as a cause of invasive pulmonary aspergillosis [40]. Therefore, identifying only this species in the study lowers the possibility of significant pulmonary infections in the school children, acquired through the handwashing process.

In relation to rotavirus, all the test results were negative. In Ghana, almost half of the diarrhoeal disease hospitalization cases of children under 5 years of age are caused by rotavirus [41]; hence, this virus was included in the microbes under investigation in the current study. The absence of this virus in the bowl water and hands of school children is a positive and a comforting finding. In spite of this, the absence of detection of rotavirus may be a reflection of the sample size used in the current study or the season when the samples were collected. It is therefore important not to infer that all handwashing bowls are free of rotavirus.

## 5. Conclusion

In the absence of running tap water in most locations, provision of “bowl water” for handwashing in preschools has been a common practice within the Accra Metropolis. The current study demonstrated the presence of microbes of faecal and zoonotic origin in some of the swabs and water samples examined (except for rotavirus). This can be of public health concern as some of the organisms identified can cause diseases especially in immunocompromised individuals and young children. Although handwashing has the ability to get rid of some microbes, this communal handwashing practice using water in a bowl could be considered a possible transmission route and may be of public concern. Even though pathogenic bacteria were isolated, the colony forming units for most of them were low. In spite of the low cfu and nondetectable rotavirus content of the water samples in the current study, adapting an improvised running water system will be an improvement. Therefore, adapting a system like the “tippy-tap” which is already practiced in some schools in Ghana and has proven to deliver promising results since its introduction in some parts of Uganda [42] is highly recommended.

## Figures and Tables

**Table 1:** Demographic data of schools recruited in the study.

School ID	Location	Ownership	Type of school
SA	Arena	Government	Preschool with primary level
SB	Arena	Private	Preschool up to JHS level
SC	Chorkor	Private	Preschool only
SD	Chorkor	Private	Preschool with primary level
SE	Mamprobi	Private	Preschool with primary level
SF	Korle Gonno	Private	Preschool with primary level

**Table 2:** Microbial contamination of different sources of bowl water and hands of children.

Microorganism	Main, *n* (%) *N* = 18	Soapy, *n* (%) *N* = 18	Rinse, *n* (%) *N* = 18	Hand before washing, *n* (%) *N* = 36	Hand after washing, *n* (%) *N* = 36
*Parasites *					
*C. parvum*	1 (12.5)	2 (7.1)	2 (9.1)	0 (0)	0 (0)
*C. cayetanensis*	0 (0.0)	2 (7.1)	2 (9.1)	0 (0)	0 (0)

*Bacteria*					
*Staphylococcus* spp.	2 (25.0)	5 (18.0)	5 (22.7)	12 (85.7)	15 (31.2)
*Salmonella* spp.	0 (0.0)	4 (14.3)	2 (9.1)	0 (0.0)	9 (18.8)
*E. coli *	2 (25.0)	3 (10.7)	3 (13.6)	0 (0.0)	8 (16.7)
*Citrobacter* spp.	1 (12.5)	3 (10.7)	3 (13.6)	0 (0.0)	6 (12.5)
*K. pneumonia*	1 (12.5)	3 (10.7)	2 (9.1)	0 (0.0)	
*K. oxytoca*	0 (0.0)	2 (7.1)	2 (9.1)	0 (0.0)	4 (8.3)
*Klebsiella* spp.	1 (12.5)	2 (7.1)	1 (4.6)	0 (0.0)	4 (8.3)
*P. mirabilis*	0 (0.0)	1 (3.6)	0 (0.0)	0 (0.0)	0 (0.0)

*Fungus*					
*A. niger *	0 (0.0)	1 (3.6)	0 (0.0)	2 (14.3)	2 (4.2)

*Virus*					
Rotavirus	0 (0.0)	0 (0.0)	0 (0.0)	0 (0.0)	0 (0.0)

*N* represents the total number of samples used. *n* represents number of particular microbes identified.

**Table 3:** Bacteria colony count of water samples (cfu/mL) and hand-swab samples (cfu/g).

Bacterial isolates	Water sources			Swabs	
	Main	Soapy	Rinse	Hand before washing	Hand after washing
*Staphylococcus* spp.	1.2 × 10^3^	1.5 × 10^3^	1.2 × 10^3^	1.8 × 10^2^	1.2 × 10^2^
*Salmonella* spp.	3.0 × 10^1^	3.2 × 10^1^	3.0 × 10^1^	1.0 × 10^1^	1.2 × 10^1^
*E. coli*	1.1 × 10^1^	1.3 × 10^1^	1.1 × 10^1^	1.7 × 10^1^	1.5 × 10^1^
*Citrobacter* spp.	3.0 × 10^1^	4.2 × 10^1^	1.4 × 10^1^	3.7 × 10^1^	3.0 × 10^1^
*K. pneumonia*	1.8 × 10^2^	2.3 × 10^2^	1.5 × 10^2^	7.0 × 10^2^	1.8 × 10^2^
*K. oxytoca*	2.3 × 10^2^	2.2 × 10^2^	2.0 × 10^2^	4.0 × 10^2^	4.2 × 10^2^
*Klebsiella* spp.	2.0 × 10^2^	1.3 × 10^2^	1.0 × 10^2^	3.3 × 10^2^	3.3 × 10^2^
*P. mirabilis*	1.5 × 10^3^	1.0 × 10^3^	3.0 × 10^3^	1.5 × 10^4^	4.3 × 10^3^

## Abbreviations

*K. oxytoca*: * Klebsiella oxytoca*
*E. coli*: * Escherichia coli*
*Citrobacter* spp.: * Citrobacter* species
*P. mirabilis*: * Proteus mirabilis*
*C. parvum*: * Cryptosporidium parvum*
*C. cayetanensis*: * Cyclospora cayetanensis*
Z-N: Ziehl-Neelsen
MA: MacConkey agar
BA: Blood agar
WHO: World Health Organization
GSFP: Ghana School Feeding Programme
CLSI: Clinical and Laboratory Standards Institute
CFU: Colony forming unit
JHS: Junior High School.
